# Optimal Therapy for Multidrug-Resistant *Acinetobacter baumannii*

**DOI:** 10.3201/eid1601.090922

**Published:** 2010-01

**Authors:** Burke A. Cunha

**Affiliations:** Winthrop-University Hospital, Mineola, New York, USA; State University of New York, Stony Brook, New York, USA

**Keywords:** Acinetobacter, colistin, XDR, A. baumannii, multidrug-resistant, colisitin, bacteria, letter

**To the Editor:** I read with interest the article by Doi et al. about a lung transplant patient presumed to have *Acinetobacter baumannii* ventilator-associated pneumonia (*1*), but some points deserve comment. *A. baumannii* is a relatively avirulent organism that frequently colonizes body fluids. For multidrug-resistant strains, antimicrobial drug selection is limited. Resolution of this patient’s pulmonary infiltrates suggests that they were not caused by *A. baumannii* that persisted in respiratory secretions. Because *A. baumannii* persisted in this patient’s respiratory secretions, colistin and cefepime were given. Colistin is an antimicrobial drug with low resistance potential; but when given by inhalation, it may lead to drug resistance (*2*,*3*).

Doi et al. stated that the patient’s *A. baumannii* strain lacked susceptibility to all available antimicrobial drugs but that cefepime and tigecycline were intermediately susceptible (MICs 16 μg/mL and 2.0 μg/mL, respectively) (*1*). Intermediate susceptibility may also be interpreted as relatively susceptible when achievable serum or tissue concentrations exceed the MIC. The article did not mention the dosages of colistin, tigecycline, and cefepime. A 2-g dose of cefepime given intravenously results in peak serum levels of ≈163 μg/mL with a relatively low volume of distribution (0.29 L/kg), which would not be expected to eradicate *A. baumannii* in respiratory secretions. High-dose intravenous tigecycline (initial dose of 200 mg followed by 100 mg daily) has been used to treat *A. baumannii*, achieving peak concentrations of ≈3 μg/mL, which exceed the isolate’s MIC of 2 μg/mL, and a high volume of distribution (8 L/kg), which would be expected to eradicate *A. baumanii* in respiratory secretions.

Optimal treatment for *A. baumannii* depends on susceptibility, pharmacokinetic principles, and site of infection. For optimal effectiveness, cefepime and tigecycline should have been given at high doses. To prevent potential resistance, antimicrobial drugs should not be given by inhalation (*3*). The alleged advantage of inhalation therapy is high local drug concentrations, but concentrations in some alveoli may be subtherapeutic (*3*). If possible, tigecycline should not be used to treat *A. baumannii* infections; however, if it is used, high doses should be given to optimize its pharmacokinetic attributes (*4*,*5*).
