# Supplementary material for: Optimal Therapy for Multidrug-Resistant Acinetobacter baumannii
Source: Emerg Infect Dis. 2010 Jan;16(1):170–1. doi: 10.3201/eid1601.090922 (PMC2874367; doi:10.3201/eid1601.090922)
Supplement: Related texts

# Letter

**DOI:** 10.3201/eid1601.091467

**Published:** 2010-01

**Authors:** David L. Paterson, Yohei Doi

**Affiliations:** University of Queensland Centre for Clinical Research, Brisbane, Queensland, Australia (D.L. Paterson); University of Pittsburgh, Pittsburgh, Pennsylvania, USA (Y. Doi)

## In Response

We welcome Burke A. Cunha’s letter (*1*) but disagree with him regarding 4 issues. First, he states that colistin has a “low resistance potential” (*1*). Although colistin has had low resistance for a long time, we are concerned that development of resistance to colistin is a growing problem. Heteroresistant *Acinetobacter* isolates are readily found (*2*). Lee et al. (*3*) recently found decreases in polymyxin B susceptibility during therapy.

Second, Cunha states, “Intermediate susceptibility may also be interpreted as relatively susceptible when achievable serum or tissue concentrations exceed the MIC” (*1*). We support the concepts that break points are artificial and that pharmacodynamic optimization may enable treatment for some organisms that are not susceptible to certain antimicrobial drugs. However, consideration of more than the MIC is necessary. Cefepime is a time-dependent bactericidal drug; therefore, effectiveness depends more on the time that the concentration of drug is above the MIC than on peak serum concentrations.

Third, Cunha states, “For optimal effectiveness, cefepime and tigecycline should have been given at high doses” (*1*). Unfortunately, in the current era of antimicrobial drug resistance, there are no “shoulds.” The high-dose tigecycline regimen that Cunha proposes for multidrug-resistant organisms has never, to our knowledge, been evaluated in randomized trials or even in large prospective evaluations. We all must admit that we do not know the optimal way to treat such infections and that we need rigorous evaluation of novel regimens. Anecdotal experience must not be translated into imperatives.

Fourth, Cunha states that “antimicrobial drugs should not be given by inhalation” (*1*). We agree that widespread use of aerosolized antimicrobial drugs cannot be recommended. However, aerosolized amikacin with a new-generation nebulizer is being tested in phase 2 clinical trials (*4*). As to the potential utility of aerosolized antimicrobial drugs, we prefer to keep an open mind pending the results of these trials.

## References

[1] Cunha B. Optimal antibiotic therapy for multidrug resistant (MDR) *Acinetobacter baumannii.* Emerg Infect Dis. 2010;16:170. 10.3201/eid1601.09092220031080PMC2874367

[2] Li J, Rayner CR, Nation RL, Owen RJ, Spelman D, Tan KE, Heteroresistance to colistin in multidrug-resistant *Acinetobacter baumannii.* Antimicrob Agents Chemother. 2006;50:2946–50. 10.1128/AAC.00103-0616940086PMC1563544

[3] Lee J, Patel G, Huprikar S, Calfee DP, Jenkins SG. Decreased susceptibility to polymyxin B during treatment for carbapenem-resistant *Klebsiella pneumoniae* infection. J Clin Microbiol. 2009;47:1611–2. 10.1128/JCM.02466-0819261795PMC2681849

[4] Luyt CE, Combes A, Nieszkowska A, Trouillet JL, Chastre J. Aerosolized antibiotics to treat ventilator-associated pneumonia. Curr Opin Infect Dis. 2009;22:154–8. 10.1097/QCO.0b013e328322a00619276883
